# Impacts of the One Big Beautiful Bill Act on African Public Health Systems

**DOI:** 10.1002/puh2.70309

**Published:** 2026-07-22

**Authors:** Majani Edward, Jusu Musa, Mlemile Stephen, Sam Soko Bundu, Caroline Nakasita Alex, Ameh Wisdom Owoicho, Naima Mbwana

**Affiliations:** ^1^ Research and Training Unit, Department of Research and Innovation MacWish College of Health and Allied Sciences Mwanza Tanzania; ^2^ Centre for Reforms, Innovation, Health Policies and Implementation Research Dodoma Tanzania; ^3^ National Public Health Agency Ministry of Health Freetown Sierra Leone; ^4^ Department of Public Health St. Francis University College of Health and Allied Sciences Ifakara Tanzania; ^5^ Federal University of Technology Minna Nigeria

## Abstract

The enactment of the One Big Beautiful Bill Act (OBBBA) in July 2025 represents a fundamental shift in United States global health financing, with significant implications for African public health systems. For more than three decades, US development assistance for health particularly through initiatives such as the President's Emergency Plan for AIDS Relief (PEPFAR), the President's Malaria Initiative, and maternal and child health programs—has been central to disease control and health system strengthening across sub‐Saharan Africa. OBBBA institutionalizes reduced funding baselines under an “America First Global Health Strategy,” constraining bilateral assistance and limiting flexible mechanisms that previously supported surveillance, laboratory systems, workforce development, and pandemic preparedness. This perspective examines the anticipated consequences of OBBBA for African public health through three interconnected lenses: disease‐specific impacts on HIV/AIDS, tuberculosis, malaria, and maternal and child health; systemic effects on health systems, including workforce sustainability, supply chains, surveillance, and research infrastructure; and broader economic and geopolitical implications for health sovereignty and partnership dynamics. Drawing on existing empirical evidence, modeling studies, and historical analogues of funding disruptions, we argue that abrupt reductions in US global health financing risk reversing hard‐won gains in disease control, exacerbating health system fragility, and increasing vulnerability to emerging infectious threats. At the same time, the policy shift may accelerate diversification of partnerships and prompt renewed debates on domestic resource mobilization and African health sovereignty. We conclude that coordinated responses by African governments, regional bodies, international institutions, and development partners are urgently required to mitigate risks, protect treatment continuity, and sustain progress toward global health security and the Sustainable Development Goals.

## 1 Introduction

The One Big Beautiful Bill Act (OBBBA, public law 119–21), enacted in July 2025, introduces a marked reorientation of United States federal spending priorities, including a recalibration of global health engagement. Historically, US development assistance for health has played a central role in shaping disease control efforts and health system infrastructure across Africa [1]. Programs, such as the President's Emergency Plan for AIDS Relief (PEPFAR)—launched in 2003, the President's Malaria Initiative—launched in 2005, and bilateral maternal and child health initiatives (which expanded significantly from 2010 onward) have contributed substantially to reductions in infectious disease morbidity and mortality [2], as well as to the strengthening of surveillance systems, laboratory networks, and health workforce capacity. The institutionalization of reduced funding baselines under OBBBA therefore represents more than a routine budgetary adjustment; it signals a structural shift with potentially far‐reaching implications for African public health [3]. The concrete provisions in OBBBA responsible for these shifts include section 301, which caps global health aid at approximately $4 billion annually (a dramatic decrease) from the historical $10–$11 billion average. Furthermore, Section 402 mandates a phased withdrawal of multilateral contributions to the Global Fund. Modeling suggests the magnitude of this impact could result in 2.5 million additional HIV infections globally by 2030 and an estimated 300,000 excess child deaths annually due to weakened maternal health and immunization networks [4]. The loss of approximately 20% of the PEPFAR‐supported health workforce (approx. 200,000 jobs) risks a total collapse of lab‐based surveillance in high‐burden regions [5].

Although this article discusses Africa broadly, it focuses primarily on sub‐Saharan Africa, as this region receives over 80% of US bilateral health assistance [4, 5]. The continental focus is however maintained to address the broader geopolitical role of the African Union (AU) in fostering health sovereignty.

For nearly three decades, US financing has underpinned large‐scale HIV treatment and prevention efforts, particularly through PEPFAR, which has supported millions of people living with HIV across sub‐Saharan Africa [4]. Similar investments have bolstered tuberculosis and malaria control, routine immunization, and maternal health services. These programs have operated not only as disease‐specific interventions but also as platforms for broader health system strengthening, enabling task‐shifting, integrated service delivery, and data‐driven decision‐making [6]. The success of these efforts has been widely documented, with measurable declines in HIV incidence, AIDS‐related mortality, and preventable child deaths.

OBBBA's policy shift occurs at a critical juncture for African health security. Many health systems remain vulnerable to antimicrobial resistance, emerging and re‐emerging infectious diseases, and climate‐related health shocks [7]. The COVID‐19 pandemic exposed persistent weaknesses in surveillance, laboratory capacity, and supply chains, even as it catalyzed recent investments to address these gaps [8]. Reductions in US bilateral assistance risk undermining these fragile gains precisely when robust, resilient systems are most needed [9].

In parallel with OBBBA, substantial reductions in USAID‐supported global health programming during 2025 further intensified concerns regarding the sustainability of health services across Africa. These reductions included the termination or suspension of numerous global health contracts and grants, workforce downsizing across implementing partners, and disruptions to technical assistance programs supporting HIV/AIDS, maternal and child health, disease surveillance, laboratory systems, and humanitarian health services. Although some funding streams and emergency health activities were subsequently restored following legal and administrative reviews, many programs continued to experience operational uncertainty, staffing shortages, and interruptions in service delivery. The resulting disruptions have affected workforce support, procurement systems, community‐based health interventions, and disease monitoring activities in several low‐resource settings [10], highlighting the vulnerability of African health systems to abrupt shifts in donor financing and policy priorities.

This article explores the anticipated implications of OBBBA for African public health across three key dimensions. First, it considers the potential effects on disease‐specific programs, including HIV/AIDS, tuberculosis, malaria, and maternal and child health. Second, it examines broader consequences for health systems, including workforce sustainability, supply chain resilience, surveillance, and research capacity. Third, it discusses the economic and geopolitical implications for health financing sustainability, national sovereignty, and partnership dynamics.

Rather than presenting a formal empirical analysis, this article offers a structured, evidence‐informed commentary drawing on existing literature, modeling studies, and historical experiences of funding disruptions. By synthesizing these insights, the article aims to inform policy dialogue and highlight priority areas for action in the context of shifting global health financing.

## 2 Implications and Challenges

### 2.1 Disease‐Specific Impacts

OBBBA‐driven reductions in US global health financing pose immediate risks to disease control programs across Africa [11]. HIV/AIDS programs are particularly vulnerable due to their reliance on PEPFAR‐supported treatment, prevention, and workforce infrastructure [12]. Funding contractions threaten antiretroviral therapy continuity through procurement shortfalls, supply chain delays, and workforce retrenchment [13]. Treatment interruptions are associated with rapid viral rebound, increased transmission risk, and the emergence of drug‐resistant HIV strains [14]. Prevention programs targeting key populations—often insufficiently supported by domestic systems—may face suspension, increasing the risk of new infections and reversing progress in stigma reduction [15].

Tuberculosis and malaria control efforts are similarly at risk due to their dependence on integrated service delivery platforms shared with HIV programs. Disruptions in HIV care can lead to increased TB incidence and mortality, particularly in settings with high TB/HIV co‐infection rates [16]. Malaria control efforts, which rely on sustained access to diagnostics and effective therapies, may experience setbacks if supply chain instability accelerates drug resistance [17]. Maternal and child health services, including routine immunization, antenatal care, and skilled birth attendance, are also vulnerable. Historical evidence shows that declines in immunization coverage are often followed by disease resurgence, disproportionately affecting rural and marginalized populations [18].

### 2.2 Health System Effects

Beyond disease‐specific programs, reductions in external financing threaten core health system functions [19]. The health workforce is particularly exposed, as international funding has long supported salaries, training, and supervision. Reduced funding may accelerate workforce attrition, especially in underserved areas, weakening service delivery and disrupting training pipelines [20].

Recent reductions in USAID‐supported programming have further amplified these vulnerabilities by disrupting externally supported service delivery platforms, technical assistance programs, and community‐based health interventions across several African countries [21]. Such disruptions may weaken institutional continuity and reduce preparedness for future outbreaks and humanitarian emergencies. Supply chain systems are also at risk, with potential increases in stock‐outs, procurement inefficiencies, and the circulation of substandard or counterfeit medicines. Surveillance and laboratory networks—critical for early outbreak detection and pandemic preparedness—may experience setbacks, reversing gains made during and after the COVID‐19 pandemic. Reduced investment in research infrastructure and international collaboration may further limit Africa's capacity to generate locally relevant evidence to guide policy and program adaptation [22]. The importance of maintaining robust surveillance and outbreak response systems has been underscored by the recent Ebola outbreak affecting the Democratic Republic of Congo and Uganda, which prompted heightened international concern and emergency response measures in 2025 [23]. The outbreak demonstrated how delays in case detection, laboratory confirmation, workforce deployment, and cross‐border coordination can rapidly escalate public health threats. In this context, reductions in external support for surveillance, laboratory infrastructure, and emergency preparedness may weaken the capacity of African countries to detect and contain future outbreaks, with implications extending beyond national borders to regional and global health security [24].

### 2.3 Economic and Geopolitical Implications

The broader economic consequences of reduced health financing are likely to be substantial [25]. Increased disease burden, workforce losses, and reduced productivity may impose long‐term costs that exceed short‐term fiscal savings. Governments may face difficult trade‐offs as they attempt to absorb funding gaps, potentially diverting resources from other critical sectors such as education and infrastructure [26].

Geopolitically, reduced US engagement is likely to accelerate the diversification of global health partnerships [27]. Although this may enhance African agency and reduce dependency on a single donor, it also introduces new challenges related to governance, quality assurance, and alignment with national priorities [28]. The evolving landscape presents both risks and opportunities, underscoring the need for strategic engagement and strengthened institutional capacity to manage partnerships effectively.

## 3 Future Directions

A coherent and sustainable response to the implications of OBBBA requires a coordinated, multi‐stakeholder strategy that strengthens health system resilience while advancing Africa's long‐term strategic autonomy. African governments, working closely with regional institutions such as the AU and Regional Economic Communities including ECOWAS and SADC, must prioritize domestic resource mobilization through progressive and innovative financing mechanisms. This includes earmarked health taxes, improved efficiency in pharmaceutical procurement, and strengthened public financial management systems to minimize wastage. Although replacing external funding entirely is not immediately feasible, gradual and targeted increases in domestic investment—aligned with commitments such as the Abuja Declaration—can safeguard essential services, including HIV, TB, malaria, and immunization programs, while supporting workforce retention and access to critical commodities. At the regional level, accelerating initiatives such as pooled procurement mechanisms and strengthening regulatory bodies like the African Medicines Agency can reduce costs, improve access to quality medicines, and promote local pharmaceutical manufacturing.

Simultaneously, African countries must pursue diversified partnerships with clear strategic intent. Engagements with emerging bilateral and multilateral partners should be guided by alignment with national health priorities, transparency, quality assurance, and commitments to technology and skills transfer. Strengthening national capacities in health diplomacy, procurement negotiation, and regulatory oversight will be essential to ensure that such partnerships reinforce, rather than compromise, health sovereignty. International actors—including the World Health Organization, UNAIDS, and the European Union—must also adapt their roles by providing time‐bound, catalytic support during this transition. This includes emergency bridge financing to maintain essential health services, investments in surveillance and laboratory systems, and support for health workforce stability, particularly in light of evolving global financing landscapes.

The private sector equally represents a critical pillar in this transition. Through public‐private partnerships, investments in local pharmaceutical production, and the expansion of digital health innovations such as mobile health platforms, private actors can enhance service delivery and system efficiency. Additionally, impact investing models offer opportunities to expand healthcare access in underserved areas, helping to offset reductions in traditional donor funding. Finally, African researchers and policy advocates must play a central role by generating locally led, context‐specific evidence on the impacts of funding transitions and identifying effective mitigation strategies. Such evidence will be essential in informing policy, strengthening advocacy, and shaping sustainable, African‐driven health financing models that are responsive to the continent's unique needs and priorities.

## 4 Conclusion

The OBBBA marks a critical inflection point for African public health, with implications that extend far beyond short‐term budgetary adjustments. By institutionalizing reductions in US global health financing, OBBBA places at risk the continuity of essential disease control programs, the stability of health workforces, and the integrity of surveillance and laboratory systems that underpin pandemic preparedness. Evidence from past funding disruptions suggests that the costs of abrupt disengagement—measured in preventable illness, mortality, and long‐term economic loss—are likely to exceed any immediate fiscal savings.

At the same time, this moment exposes structural vulnerabilities created by prolonged reliance on external financing and underscores the urgency of building more resilient, self‐directed health systems. The path forward will be uneven across countries, shaped by domestic fiscal capacity, governance strength, and the ability to mobilize alternative partnerships. Nevertheless, coordinated action by African governments, regional bodies, international institutions, and development partners can prevent the erosion of hard‐won gains and protect the most vulnerable populations.

Ultimately, global health security is indivisible. Investments in African health systems contribute not only to national well‐being but also to global disease prevention and preparedness. How African nations and the international community respond to OBBBA will determine whether this policy shift becomes a catalyst for regression—or an impetus for a more sustainable, equitable, and resilient global heal.

## Author Contributions

**Majani Edward**: conceptualization, supervision, writing – original draft, writing – review and editing, investigation. **Jusu Musa**: conceptualization, investigation, writing – original draft. **Mlemile Stephen**: investigation. **Sam Soko Bundu**: writing – original draft. **Caroline Nakasita Alex**: writing – original draft. **Ameh Wisdom Owoicho**: writing – original draft. **Naima Mbwana**: writing – original draft.

## Funding

The authors have nothing to report.

## Ethics Statement

With respect to this type of article, we didn't seek informed consent we used existing data from the prior findings.

## Consent

Data were collected from the prior findings, so no informed consent was employed.

## Conflicts of Interest

The authors declare no conflicts of interest.

## Transparency Statement

The lead author Majani Edward affirms that this manuscript is an honest, accurate, and transparent account of the study being reported; that no important aspects of the study have been omitted.

## Data Availability

The authors confirm that the data supporting the findings of this study are available within the article.
